# Patient Satisfaction with Advanced Medical Technology-Based Treatments in Integrative Medicine: A Structural Equation Modeling Approach Among Patients with Chronic Diseases

**DOI:** 10.3390/healthcare14111485

**Published:** 2026-05-27

**Authors:** Prajisteanu Constantin-Bogdan, Anamaria-Cătălina Radu, Georgeta Ionescu, Daniel Diaconescu, George Mihail Man

**Affiliations:** 1 Department of Marketing and Medical Technology, University of Medicine and Pharmacy “Carol Davila” Bucharest, 020021 Bucharest, Romania; constantin-bogdan.prajisteanu@drd.umfcd.ro; 2 Romanian Academy, Institute of National Economy, 050711 Bucharest, Romania; anamaria_radu15@yahoo.com; 3 Department of Medical Assistance and Physical Therapy, Faculty of Sciences, Physical Education and Informatics, University Center of Pitești, National University of Science and Technology Politehnica Bucharest, 110040 Pitești, Romania; george_mihail.man@upb.ro

**Keywords:** integrative and complementary medicine, patient satisfaction, advanced medical technologies, chronic disease management, healthcare innovation

## Abstract

**Background:** Integrative and complementary medicine has gained increasing recognition as a patient-centered approach to managing chronic conditions, particularly through the integration of advanced medical technologies into conventional care. These approaches aim to enhance treatment effectiveness, improve patient experience, and support long-term health outcomes, while promoting a more holistic and personalized model of healthcare delivery. **Methods:** This study employs a cross-sectional quantitative research design based on a sample of 159 patients who underwent treatments involving advanced medical technologies integrated into integrative medicine protocols. Data was collected using a structured questionnaire and analyzed using a structural equation modeling approach to assess the relationships between key determinants and patient satisfaction. **Results:** The findings indicate that patient satisfaction is significantly associated with perceived benefits and trust, while prior experience and perceived costs exert weaker effects. In contrast, perceived outcomes and service quality did not show statistically significant associations with patient satisfaction within the analyzed sample. The independent variables included in the model explained 69% of the variance in patient satisfaction. Furthermore, patient satisfaction explained 42% of the variance in patients’ intention to reuse such treatments. **Conclusions:** The patients included in the study reported high levels of satisfaction with advanced medical technology-based treatments in integrative medicine. The results highlight the importance of perceptual factors, particularly perceived benefits and trust, in shaping patient satisfaction within integrative care settings. These findings may support the integration of advanced medical technologies into patient-centered healthcare models and contribute to the ongoing development of innovative and sustainable practices in integrative and complementary medicine.

## 1. Introduction

Integrative medicine is currently regarded as a modern and complex approach to healthcare. Its role is to combine the established methods of conventional medicine with therapies specific to complementary medicine [1], such as Acupuncture, Ayurveda, Yoga, or Naturopathy [2]. This form of medicine is not limited to treating diseases or the symptoms experienced by the patient; rather, it aims to care for the individual in their entirety [3]. Within integrative medicine, the human being is viewed as a whole, and the applied procedures target the body, mind, and spirit [4]. In recent years, increased attention has been directed toward the integration of innovative and technology-supported interventions within integrative medicine protocols, particularly in the context of chronic disease management and patient-centered healthcare.

The objectives of integrative medicine include combining techniques from conventional medicine, which are effective in treating acute conditions with potentially life-threatening risks, with procedures specific to complementary medicine that can contribute to alleviating pain and symptoms associated with chronic diseases [5]. The concept of integrative medicine emerged in the scientific literature in the early 1990s and is currently associated with patient-centered, scientifically grounded, and interdisciplinary healthcare approaches [6,7]. Integrative medicine emphasizes the physician–patient relationship, individualized care, and the use of scientifically grounded interventions focused on the patient as a whole [7,8,9,10,11]. Another distinguishing element of integrative medicine is its high level of flexibility regarding treatment options, an approach complemented by the holistic evaluation of the patient. This perspective allows for the development of personalized therapeutic plans based on the patient’s medical history, the etiology of the disease, and the patient’s lifestyle [12]. A fundamental aspect of integrative medicine is prevention, with emphasis on reducing the risk of disease occurrence and maintaining long-term health.

### 1.1. Applicability of Integrative Medicine in the Management of Chronic Conditions

In recent years, integrative medicine has experienced significant growth among patients who use it to treat various chronic conditions. It can be applied either as a standalone treatment method or as a complementary approach alongside other forms of care, such as holistic medicine, with the aim of achieving faster and more effective outcomes [13]. Currently, integrative medicine is used in the management of a wide range of chronic diseases, including cardiovascular conditions [14,15], neurological disorders [16], psychiatric conditions [17,18], metabolic diseases [19], autoimmune and inflammatory disorders [20], musculoskeletal conditions [21], gastrointestinal diseases [22], oncological conditions [6], and respiratory diseases such as asthma [23]. Moreover, recent research has highlighted its benefits in the treatment of certain chronic diseases affecting children [24], as well as its applicability in the field of plastic surgery [25,26].

For the management of different chronic conditions, integrative medicine employs a wide range of therapies, including acupuncture, phytotherapy, meditation, and yoga techniques [27]. The main objective of these interventions is to reduce pain and alleviate the symptoms associated with chronic diseases. In recent years, numerous studies have highlighted the added value that this therapeutic approach provides to patients, regardless of the type of condition [28]. Specialists in the field have emphasized that, when developing personalized medical procedures for the treatment of chronic diseases, the connection between the patient’s mind and body must be carefully analyzed. Therefore, within the therapeutic process, it is essential to understand how emotional factors may have contributed to the onset or exacerbation of a disease [29,30]. Social support also plays an important role in the recovery process of patients with chronic diseases [31].

When analyzing the contribution of integrative medicine to the treatment of chronic conditions in pediatrics, it has been observed that specialists primarily aim to identify the underlying causes that led to the onset of the disease [32]. Therapeutic interventions take into account the child’s lifestyle, sleep quality, and the level of stress within the family environment. For this reason, treatment protocols often include a balanced diet, appropriate sleep hygiene practices, stress-reduction techniques, and conventional medical procedures. In addition, integrative medicine protocols for children frequently include breathing exercises and mindfulness practices [24].

Regarding the application of integrative medicine in oncology, there are currently a number of procedures and treatments aimed at improving tolerance to the side effects of oncological therapies and enhancing patients’ quality of life [33]. Integrative medicine is also increasingly used in the field of psychiatry for the management of chronic disorders. The treatments and procedures specific to these conditions aim to reduce symptoms and contribute to improving the overall condition of patients [34]. In this context, integrative medicine plays an important role in preventing the onset of psychiatric disorders and identifying the deeper emotional causes that may have influenced the development of the disease. In psychiatric disorders, integrative medicine emphasizes prevention, holistic care, and patient involvement in the therapeutic process [35].

### 1.2. Advanced Medical Technologies Used in Integrative Medicine Protocols for the Treatment of Chronic Conditions

Integrative medicine has been used for many years in the treatment of various chronic conditions. Technological progress and the development of new technologies in this field have led to the emergence of modern devices based on advanced treatment techniques for different diseases. The new medical technologies that have recently emerged play an essential role in optimizing treatments for chronic diseases. Some of the most well-known advanced technologies currently used for their treatment include PAPIMI [36], LLLT [37], oxygen–ozone therapy [38], and real-time neurofeedback [39]. These technologies are increasingly associated with innovative and patient-centered healthcare approaches aimed at improving treatment effectiveness, patient experience, and long-term health outcomes.

PAPIMI therapy is based on a high-intensity pulsed electromagnetic field. It is included in integrative medicine protocols to stimulate tissue regeneration, reduce inflammation associated with chronic conditions, and alleviate muscular or joint pain [40]. Previous studies demonstrated its effectiveness in musculoskeletal recovery, inflammation control, and the management of several chronic conditions [41,42,43,44,45]. Its role is to accelerate cellular healing by stimulating mitochondrial activity. This practice provides very good results in the treatment of chronic conditions, reducing inflammation in the body, accelerating tissue regeneration, and alleviating pain [46]. In recent years, LLLT has been significantly improved and is now used in the treatment of chronic pain [47,48], which is frequently associated with conditions such as carpal tunnel syndrome, arthritis, or tissue injuries [49]. LLLT is also used in wound healing [50]. Some studies [51] have shown that this advanced medical technology, when integrated into integrative medicine protocols, is effective in treating shoulder impingement syndrome, providing analgesic, anti-inflammatory, and regenerative effects.

Another advanced technology used within integrative medicine protocols is oxygen–ozone therapy, which includes essential procedures for the treatment of chronic conditions [52]. Specialized studies [38] have shown that ozone therapy is particularly important in the treatment of chronic non-healing wounds, such as diabetic ulcers, pressure ulcers, and venous ulcers. Other studies highlighted that oxygen–ozone therapy contributes to reducing inflammation, improving microcirculation, decreasing oxidative stress, and supporting tissue repair processes [53,54,55,56]. Real-time neurofeedback is an advanced medical technology used in integrative medicine protocols for monitoring and regulating brain electrical activity through operant learning mechanisms. Specialists frequently use this technology to treat chronic conditions such as anxiety disorders, ADHD, sleep disorders, or chronic pain [39]. Previous studies demonstrated that neurofeedback contributes to pain reduction, improved quality of life, and better management of neurological and psychiatric disorders [57,58,59,60].

Although previous studies have investigated the clinical applications of advanced medical technologies in integrative medicine, limited research has explored how patients evaluate these treatments from the perspective of satisfaction, perceived benefits, trust, and intention to reuse such therapies in the future.

### 1.3. Theoretical Background and Conceptual Framework

The increasing use of advanced medical technologies in the field of integrative medicine has generated growing interest among specialists investigating this area, particularly in identifying the factors that influence patient satisfaction with these treatment protocols. In recent years, researchers and healthcare professionals have also become increasingly interested in understanding how the level of satisfaction experienced by patients may influence their loyalty toward such treatments and their intention to continue using them in the future. Previous studies conducted in the healthcare field [61] have shown that one of the most important factors influencing patient satisfaction with medical services is represented by the outcomes obtained following the treatment process. Patients evaluate healthcare services based on both service quality and treatment outcomes. In addition, AlQudah et al. [62] highlighted that the acceptance of new medical technologies has a strong influence on patients’ perceptions regarding the usefulness and effectiveness of benefits obtained through such treatments. Therefore, perceived usefulness and value are considered among the most important factors capable of generating positive attitudes toward advanced medical technologies. Other studies conducted in this field [63] demonstrated that patients tend to experience higher levels of satisfaction when the benefits obtained from medical procedures exceed their initial expectations.

Another important factor that may significantly influence patient satisfaction is represented by prior patient experience. Previous interactions with advanced medical technologies or with treatments based on such technologies may strongly influence both patients’ perceptions and their level of satisfaction regarding these healthcare services [64]. Leonardsen et al. [65] argued that positive previous experiences contribute to increasing patients’ trust, making them feel more comfortable when using these technologies. As a result, this contributes to higher levels of satisfaction following the use of such medical services. Trust is also considered an important determinant of patient satisfaction regarding advanced medical technologies integrated into integrative medicine protocols. Previous studies [66,67] have shown that trust both in the healthcare system and in advanced medical technologies significantly increases the acceptance of these treatments among patients.

Service quality continues to be viewed as one of the most important factors influencing patient satisfaction. Research based on the SERVQUAL model [68,69,70] has shown that factors such as effective communication, responsiveness, professionalism, empathy, and other service-related dimensions may strongly influence the way patients evaluate their healthcare experiences. Within integrative medicine, this aspect becomes particularly important because treatment protocols are usually personalized according to the needs and preferences of each individual patient. Treatment cost is also considered an important factor influencing patient satisfaction. Studies have shown that patients evaluate not only the medical experience itself, but also the balance between quality and cost. When analyzing the cost of a treatment, patients usually assess it in relation to the results obtained following the procedures [71]. Therefore, when individuals perceive that the cost of treatment is justified by the quality and benefits provided, the level of satisfaction experienced after using such treatments tends to be significantly higher [72]. In addition to the factors influencing patient satisfaction with healthcare services, previous studies have shown that satisfaction itself may further influence patients’ intention to re-use such treatments in the future. Research conducted in this field demonstrated that the more satisfied patients are with the services received, the more likely they are to continue using them and to recommend them to other individuals [69,73,74].

Considering the increasing number of patients who resort to integrative medicine protocols, as well as the growing number of studies conducted in this field, we considered it necessary to conduct a quantitative study aimed at identifying the level of satisfaction among patients with chronic diseases regarding treatments based on advanced medical technologies integrated into integrative medicine protocols. Therefore, the main objective of this study was toexamine the factors associated with patient satisfaction experienced by patients undergoing such treatments, as well as the manner in which this satisfaction may, in turn, influence patients’ intention to use such treatments again in the future in order to treat either the same condition or a new medical condition. The SEM developed in this study was based on the following hypotheses:

**H1.**  *The results obtained by patients with chronic diseases following treatments based on advanced medical technologies integrated into integrative medicine protocols are positively associated with the level of satisfaction experienced by patients* [61].

**H2.**  *Thebenefits perceived by patients following treatments based on advanced medical technologies integrated into integrative medicine protocols are positively associated with the level of satisfaction experienced by patients* [62,63].

**H3.**  *Patients’ prior experience with treatments based on advanced medical technologies integrated into integrative medicine protocols is positively associated with the level of satisfaction experienced by patients* [64,65].

**H4.**  *Patients’trust in treatments based on advanced medical technologies integrated into integrative medicine protocols is positively associated with the level of satisfaction experienced by patients* [66,67].

**H5.**  *Thequality of services provided within treatments based on advanced medical technologies integrated into integrative medicine protocols is positively associated with the level of satisfaction experienced by patients* [68,69,70].

**H6.**  *Theperceived cost of treatments based on advanced medical technologies integrated into integrative medicine protocols is positively associated with the level of satisfaction experienced by patients* [71,72].

**H7.**  *Thesatisfaction experienced by patients following treatments based on advanced medical technologies integrated into integrative medicine protocols is positively associated with their intention to use such treatments again in the future to treat the same condition or a new medical condition* [69,73,74].

## 2. Materials and Methods

### 2.1. Survey Design

The study conducted within this research was carried out between January and March 2026. The research was conducted on a sample of 159 respondents. For the purpose of data collection, a questionnaire consisting of 25 questions was developed. The study was conducted within a single private clinic specializing in integrative medicine located in Bucharest, Quantum Integrative Medicine Clinic. Given the limited number of clinics offering these technologies in Romania, most respondents came from urban areas. This aspect may limit the variability of certain perceptions and reduce the generalizability of the findings. The questionnaire was distributed among patients with chronic diseases who had undergone at least one treatment based on advanced medical technologies integrated into integrative medicine protocols. After completing the treatment procedures, some patients had the opportunity to complete the questionnaire directly within the clinic using a tablet. In addition, the questionnaire was also distributed online by email to patients from the clinic database who had previously undergone at least one treatment based on advanced medical technologies integrated into integrative medicine protocols.

Regarding the structure of the questionnaire, it should be noted that the first question was a screening question, through which only individuals belonging to the target population were selected, namely patients who had undergone at least one treatment based on advanced medical technologies integrated into integrative medicine protocols. Subsequently, the questionnaire included questions aimed at identifying respondents’ level of knowledge regarding such treatments, the sources of information they used for documentation, as well as the types of treatments they had followed up to that point. In the first part of the research, several questions were also included to identify the chronic condition from which the patients suffer, the period since they had been diagnosed, and the duration of the treatment they had followed. Subsequently, a series of questions were introduced to examine how the independent variables considered in the analysis influence the dependent variable, namely the satisfaction of patients with chronic diseases regarding the treatments based on advanced medical technologies integrated into integrative medicine protocols that they had undergone.

In the final part of the questionnaire, several questions were included in order to determine patients’ intention to recommend, in the future, treatments based on advanced medical technologies used within integrative medicine protocols to friends and relatives, as well as to identify the aspects they would seek to improve in relation to these treatments. The last section of the questionnaire included demographic questions, which were used to develop the demographic profile of the respondents.

Regarding the development of the constructs included in the SEM, it should be specified that the questionnaire items were adapted from the specialized literature and reformulated in accordance with the objectives and context of the present study. The first construct (patient-reported treatment outcomes) was measured using 4 items, the second construct (perceived benefits) using 5 items, the third construct (patients’ prior experience) using 4 items, trust using 5 items, service quality using 6 items, and perceived cost using 4 items. In addition, patient satisfaction and intention to reuse the treatments were each measured using one item. As a result, the reliability analysis was conducted on a total of 30 measurement items. Within the SEM model, the independent variables were treated as formative constructs because the items included different aspects related to patients’ perceptions of the treatments analyzed. Patient satisfaction and intention to reuse the treatments were treated as reflective constructs, as they reflect the overall perception and behavioral intention of the respondents.

Thus, it can be observed that the independent variables included in the proposed conceptual model were treated as formative constructs. This decision was made because the measurement items represented distinct dimensions that together define each construct. When analyzing the model, the indicators were not considered interchangeable manifestations of an underlying latent variable. Instead, they were viewed as complementary components. Their role was to contribute to the formation of the construct itself. Variables such as perceived benefits, trust, service quality, or perceived cost were evaluated through different aspects related to patients’ perceptions of the treatment protocols used in the study. Therefore, based on previous studies from the specialized literature, formative measurement models are considered appropriate when the indicators collectively form the construct rather than simply reflect it [75,76].

From the perspective of questionnaire development, several stages were followed. In order to ensure that the research instrument was properly constructed, the questionnaire was initially pretested on a sample of 14 respondents. The pretesting stage aimed mainly to identify unclear questions, ambiguous formulations, and possible difficulties in understanding the items included in the questionnaire. Following this process, several questions were reformulated in order to improve clarity and readability before the final administration of the questionnaire. After the final version of the questionnaire was developed, it was uploaded to the Google Forms platform and made available to respondents. After data collection, the database was downloaded, cleaned, and subsequently analyzed using the IBM SPSS 31.0 software. The testing of the model was performed using the WarpPLS 8.0 software. WarpPLS was selected because the study had an exploratory character and aimed primarily to examine predictive relationships between constructs. In addition, PLS-SEM is considered appropriate for exploratory research models and moderate sample sizes.

With regard to the types of scales used in the study, both nominal scales and semantic differential scales were employed. The questions underlying the conceptual model were measured using a 7-point Likert scale (ranging from “Strongly disagree” to “Strongly agree”). The questionnaire included 25 main questions. However, some questions contained multiple measurement items related to the latent constructs included in the SEM. For this reason, the reliability analysis was conducted on a total of 30 measurement items. The analysis of the Cronbach’s Alpha coefficient (Table 1) indicates a high level of internal consistency of the scale employed. The obtained value (α = 0.954) exceeds the recommended threshold of 0.9, demonstrating excellent reliability of the measurement instrument. This result suggests that the items included in the study consistently measure the same latent construct and exhibit a strong degree of internal correlation. Moreover, the minimal difference between the standard Cronbach’s Alpha and the value based on standardized items (0.957) indicates relatively uniform variability among the items and the absence of significant scaling discrepancies. This finding further supports the robustness of the scale and its suitability for subsequent analyses.

The assessment of the measurement model was carried out by analyzing the reliability and validity of the constructs included in the SEM. In this regard, the following indicators were examined for each latent variable included in the model: Composite Reliability (CR), Cronbach’s Alpha, Average Variance Extracted (AVE), and Full Collinearity VIF.

The results obtained from this analysis indicated a good level of internal consistency, as all Composite Reliability and Cronbach’s Alpha values exceeded the recommended threshold of 0.70. In addition, all AVE values were higher than 0.50 for the analyzed constructs. This confirms the existence of convergent validity. The Full Collinearity VIF values were below the recommended threshold of 3.3, indicating the absence of significant collinearity issues within the proposed model (Table 2).

### 2.2. Proposed SEM

Within the quantitative study conducted, a Structural Equation Model (SEM) was developed in order to identify how a series of independent variables influence the dependent variable, namely the satisfaction of patients with chronic diseases regarding treatments based on advanced medical technologies integrated into integrative medicine protocols that they have used. Within the model, the following independent variables were considered: the results obtained by patients following the treatments undertaken, the perceived benefits associated with these treatments, patients’ previous experience with such treatments, the level of trust patients place in these treatments, the quality of the services provided, and the perceived cost.

**Figure 1:**
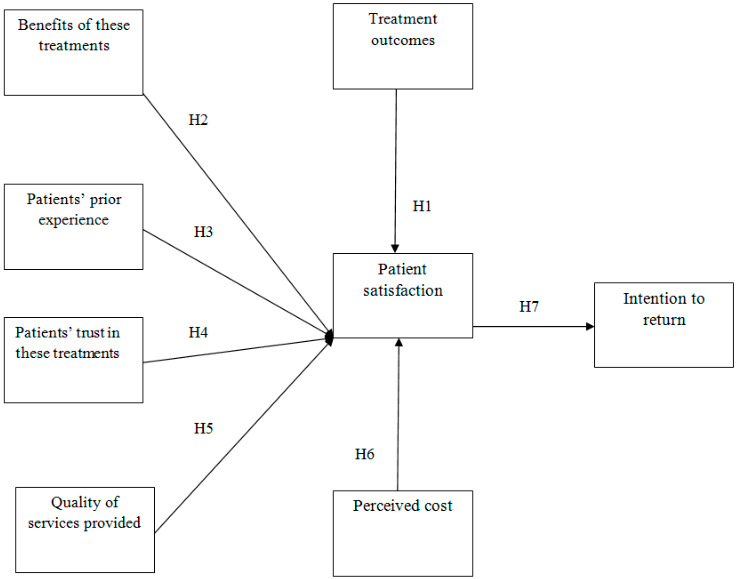
Proposed conceptual model.

In addition, the proposed conceptual model (Figure 1) aimed to identify the extent to which patients’ satisfaction with these treatments influences their intention to use such treatments again in the future should they need to treat the same condition or a new medical condition. The conceptual model proposed in this study can be observed in the figure below. At this level, the seven hypotheses previously presented can also be identified.

## 3. Results

A total of 167 respondents were initially included in the present study. Of these, 159 met the eligibility criterion established through the screening question, namely having used, prior to the time of assessment, at least one treatment based on advanced medical technologies integrated into integrative medicine protocols for the management of a chronic condition. Consequently, the statistical analysis was conducted exclusively on this sample of 159 patients.

### 3.1. Respondents’ Profile

With regard to the respondents’ profile, it can be observed that 35.8% reported having used, for the first time, treatments based on advanced medical technologies integrated into integrative medicine protocols in 2025. Additionally, 25.2% began using these therapies in 2024, 13.8% in 2022, and 13.2% in 2023. Furthermore, 11.9% of respondents indicated that they had resorted to such treatments four years ago or earlier. In terms of information sources regarding these therapies, nearly half of the respondents (49.7%) identified medical recommendations as the primary source of information. Other sources include recommendations from friends (14.5%), information obtained from acquaintances and family members (9.4%), self-directed research (8.8%), and online sources (5%). A relatively small proportion (3.1%) reported that they became aware of these treatments through promotional activities.

The analysis of the respondents’ demographic profile indicates that 61.6% of the participants were female, while 38.4% were male. From the perspective of age group distribution, 28.3% of respondents fall within the 46–55 age range, 27% are aged 36–45, 19.5% are between 56–65 years old, 9.4% are over 65, 8.8% are aged 26–35, and 6.9% are between 18–25 years old. Regarding the level of education, 48.4% of respondents hold a bachelor’s degree, 23.9% have completed high school education, 22% possess a master’s degree, and 5% have completed doctoral studies or MBA programs. Only 0.6% of participants reported not having completed any level of education. The analysis of occupational status revealed that the largest proportion of respondents (32.1%) consists of professionals with higher education. Additionally, 21.4% are business owners or administrators, 13.8% hold management positions (managers or directors), 7.5% are retirees, and 6.9% are self-employed (independent professionals). From an income perspective, 31.4% of respondents reported a monthly net income exceeding RON 10,000. Other relevant categories include 17% with incomes between RON 7001–8500, 15.7% between RON 4001–5500, 13.2% between RON 8501–10,000, and 11.3% between RON 5501–7000. Furthermore, 7.5% of respondents have incomes ranging from RON 2574–4000, while 3.8% report monthly net incomes below RON 2574. In terms of residence, the vast majority of respondents (96.9%) are from urban areas, while only 3.1% reside in rural areas.

### 3.2. Research Results

The analysis of chronic conditions for which respondents used treatments based on advanced medical technologies integrated into integrative medicine protocols highlights a diverse distribution of addressed pathologies. Thus, 38.4% of patients reported resorting to these treatments for gastrointestinal conditions, such as irritable bowel syndrome (IBS), inflammatory bowel diseases (Crohn’s disease and ulcerative colitis), gastroesophageal reflux disease (GERD), or hepatic steatosis. Additionally, 35.2% of respondents used these interventions for the management of autoimmune and inflammatory diseases, including Hashimoto’s thyroiditis, rheumatoid arthritis, lupus, psoriasis, celiac disease, and ankylosing spondylitis. At the same time, 23.3% reported using these treatments for metabolic conditions, such as type 2 diabetes mellitus, obesity, dyslipidemia, or metabolic syndrome.

Regarding other categories of conditions, 18.2% of respondents used these therapies for musculoskeletal disorders (e.g., osteoarthritis, osteoporosis, chronic low back or cervical pain, and fibromyalgia), while 8.8% used them for oncological and respiratory conditions (such as bronchial asthma, chronic obstructive pulmonary disease, or sleep apnea). Furthermore, 7.5% of patients indicated the use of these treatments for psychiatric conditions, including major depressive disorder, generalized anxiety disorder, bipolar disorder, and sleep disorders. A proportion of 6.9% of respondents used these interventions for cardiovascular diseases, such as hypertension, coronary artery disease, heart failure, or arrhythmias, while 1.3% used them for chronic dental conditions (e.g., periodontitis or untreated dental caries). In addition, 0.6% of respondents resorted to these treatments for sensory conditions, such as cataracts or age-related macular degeneration. Moreover, 8.2% of participants reported using these therapies for other types of conditions, including various dermatological disorders, immune system support, reduction in chronic inflammation, treatment of food intolerances, prevention of hair loss, and for preventive purposes.

The analysis of the time elapsed since the diagnosis of the chronic condition treated through integrative medicine therapies revealed that 33.3% of respondents were diagnosed 1–2 years ago. Additionally, 22.6% of patients reported a period of 3–4 years since diagnosis, while 18.9% stated that they had been diagnosed less than one year ago. Furthermore, 13.2% of respondents indicated that the diagnosis had been established more than 6 years ago, whereas 11.9% reported a time interval of 5–6 years since diagnosis.

The analysis of advanced medical technologies used within integrative medicine protocols for the treatment of chronic conditions highlights the predominance of certain types of interventions. Thus, 82.9% of respondents indicated the use of PAPIMI technology, while 70.3% reported oxygen–ozone therapy as part of the therapeutic regimens followed. Additionally, 34.2% of patients reported the use of low-level laser therapy (LLLT), whereas only 7.6% stated that they had benefited from real-time neurofeedback therapy. Furthermore, 26.6% of respondents indicated the use of other complementary technologies or interventions, such as Heckel thermotherapy, HHO technology, Tecar therapy, probiotic administration, various types of massage, therapeutic infusions, or other forms of thermotherapy.

The analysis of treatment duration indicates that 42.8% of patients underwent therapies for a period of 2–3 months, while 23.9% reported a duration of approximately one month. Furthermore, 16.4% of respondents stated that the treatment lasted 4–5 months, 9.4% reported durations exceeding 7 months, and 7.5% indicated a duration of 6–7 months. Regarding the number of sessions performed, 39% of respondents underwent more than 15 procedures, 26.4% benefited from 6–10 procedures, 22.6% underwent 11–15 procedures, and 11.9% participated in 1–5 procedures. In terms of the perceived balance between conventional medicine and complementary interventions within the treatment, respondents indicated a high level, with a mean score of 4.38, suggesting a harmonious integration of the two therapeutic approaches.

With regard to recommendation intention, all 159 respondents stated that they would definitely recommend these treatments based on advanced medical technologies, used within integrative medicine protocols, to close acquaintances. The analysis of aspects that could be improved reveals that 44.3% of respondents would like access to additional information regarding the functioning of the technologies used. Moreover, 35.4% mentioned the need to diversify treatment types, 30.4% would opt for expanding the range of equipment used, and 22.8% expressed a desire for more effective communication with medical staff. A small proportion, 5.1%, stated that they would not change any aspect of the treatments in their current form.

The analysis revealed a significant adoption of treatments based on advanced medical technologies integrated into integrative medicine protocols, particularly among urban patients with a high level of education and above-average income. The results indicate that, although some patients have been using these treatments for a relatively short period of time, both the level of acceptance and patient satisfaction are high. Furthermore, it was observed that these interventions are currently being used to treat a wide range of chronic conditions. The duration of treatments varies depending on the type of intervention and the complexity of the pathology addressed. Overall, the findings suggest the need to improve access to information, as well as to diversify the available therapeutic options, in order to optimize patient experience and outcomes.

### 3.3. Testing of the Structural Equation Model

**Figure 2:**
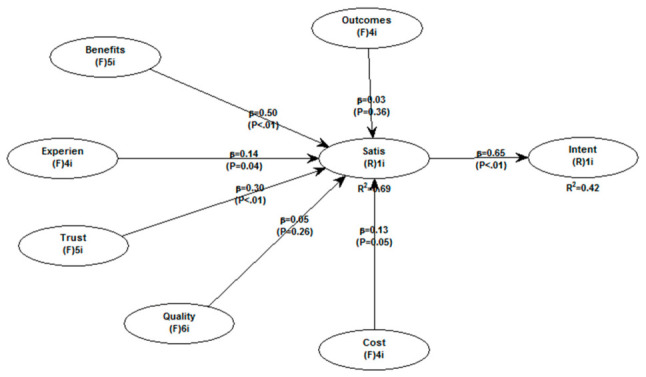
Model of patient satisfaction with chronic conditions regarding treatments based on advanced medical technologies integrated into integrative medicine protocols.

To test the proposed conceptual model, the WarpPLS software (version 8.0) was employed. This tool is widely used in structural equation modeling based on the PLS-SEM approach, enabling the simultaneous assessment of relationships between latent variables and their associated indicators. The use of this software facilitated both the testing of research hypotheses and the estimation of causal relationships among the constructs included in the model. Within the scope of this study, the analysis aimed to examine how a set of independent variables, namely, patient-reported treatment outcomes, perceived benefits, prior experience with such interventions, level of trust in treatments, quality of the services provided, and perceived cost, influence the dependent variable, namely patient satisfaction with advanced medical technology-based treatments integrated into integrative medicine protocols for chronic conditions. Furthermore, the conceptual model investigated the relationship between patient satisfaction and their intention to use such treatments in the future, should the need arise to address either the same condition or a new pathology (Figure 2). Following the estimation of the proposed conceptual model, the following results were obtained:

At the level of the proposed conceptual model, seven hypotheses were formulated and tested using the WarpPLS software. The statistical significance of the relationships included in the SEM was assessed using two-tailed tests. The Table 3 presents the hypotheses validation results, together with the β coefficients and *p*-values obtained from the model estimation. The results indicate that five hypotheses are empirically supported, while two are not supported. The first hypothesis is not supported, as the analyzed relationship is not statistically significant (β = 0.03, *p* = 0.36), indicating a negligible association between perceived outcomes and satisfaction. One possible explanation for the non-significant relationship between patient-reported outcomes and satisfaction may be that patients evaluated their treatment experience not only based on the clinical results obtained, but also based on factors such as trust and the perceived benefits of the treatments. The second hypothesis is statistically supported (β = 0.50, *p* < 0.01), revealing a strong and positive association between perceived benefits and satisfaction. This result suggests that higher levels of perceived benefits are associated with higher levels of satisfaction.

The third hypothesis is also supported (β = 0.14, *p* = 0.04), although the association is relatively weak. Thus, prior experience is positively associated with satisfaction but does not represent a key associated factor. The fourth hypothesis is statistically supported (β = 0.30, *p* < 0.01), indicating a moderate association between trust and satisfaction, highlighting the importance of trust in relation to patient satisfaction. The fifth hypothesis is not supported (β = 0.05, *p* = 0.26), as the relationship is not statistically significant, despite its positive direction. Although previous studies reported a strong relationship between service quality and patient satisfaction, this relationship was not statistically significant in the present study. One possible explanation is that, when patients use these treatments, they may place greater importance on the perceived benefits of the therapies and on the trust they have in the clinic and the procedures performed. In this context, service quality may be perceived as part of these broader dimensions. In addition, when patients choose such treatments, service quality may not be considered as important as the other factors analyzed in the study.

The sixth hypothesis showed only marginal statistical significance (β = 0.13, *p* = 0.05). Although the relationship between perceived cost and patient satisfaction was positive, the association was relatively weak and should be interpreted with caution. Additional information regarding the SEM results was examined in WarpPLS, including standard errors and effect sizes for this relationship. In the case of H6, the relationship between perceived cost and patient satisfaction showed a standard error of 0.077 and a relatively small effect size of 0.079.Finally, the seventh hypothesis is statistically supported (β = 0.65, *p* < 0.01), demonstrating a strong positive association between satisfaction and intention. This finding highlights the role of satisfaction in relation to patients’ intention to return.

The analysis of the coefficient of determination (R^2^) highlights the model’s ability to explain the variance of the dependent variable through the independent variables. In this case, the results indicate that patient satisfaction among individuals with chronic conditions regarding treatments based on advanced medical technologies, integrated into integrative medicine protocols, is explained to a proportion of 69% by the independent variables included in the model. This finding suggests a good overall fit of the proposed model. Regarding patients’ intention to return, this is explained to a proportion of 42% by the level of satisfaction experienced by patients, indicating a moderate relationship between the two variables. The Q^2^ coefficients obtained for the endogenous variables were above zero (Q^2^ satisfaction = 0.600; Q^2^ intention = 0.445), indicating good predictive relevance of the proposed model (Table 4).

An essential aspect in evaluating the validity of the proposed conceptual model is the analysis of model fit indices. The Table 5 presents the main indicators obtained from the model testing. The APC value is 0.257 and is statistically significant (*p* < 0.001), indicating that, on average, the relationships between the variables included in the model are meaningful and significantly different from zero. This result suggests a good explanatory capability at the level of the structural model. The ARS value is 0.558 (*p* < 0.001), indicating that the model explains approximately 55.8% of the variance in the dependent variables, reflecting a good level of explanatory power. Similarly, the AARS value is 0.550 (*p* < 0.001), which is close to the ARS value, suggesting that the model is not overfitted and that the results are stable and robust. Regarding collinearity, the AVIF value is 1.995, below the recommended threshold of 3.3, indicating the absence of collinearity issues at the level of indicator blocks. Furthermore, the AFVIF value is 2.250, confirming the lack of collinearity at the full model level, thus supporting proper model specification. In addition, the Full Collinearity VIF values generated by the WarpPLS software were below the recommended threshold of 3.3 for all constructs included in the model, indicating the absence of significant multicollinearity issues within the proposed conceptual model. The GoF value is 0.664, which exceeds the recommended threshold of 0.36, suggesting an acceptable overall model fit according to the criteria proposed in the WarpPLS literature. This result reflects both a good quality of the measurement model and an adequate capacity to explain structural relationships. Moreover, the values of the SPR, RSCR, SSR, and NLBCDR indices are all equal to 1, confirming the consistency of the relationships within the model, the absence of contradictory effects among independent variables, the stability of the estimated relationships, and the correct specification of causal directions, including in the context of nonlinear relationships. An important aspect that should be mentioned is that the reported fit indices (SPR, RSCR, SSR, and NLBCDR) are specific validation indicators generated by WarpPLS. These indicators are commonly used within this software environment to assess model quality and causal consistency and are generally employed in WarpPLS for model validation purposes.

## 4. Discussion

In recent years, integrative medicine has begun to gain increasing importance in the treatment of chronic conditions, as it has the capacity to combine the approaches of conventional medicine with complementary therapies. In integrative medicine, specialists do not study only the conditions patients suffer from, but also the factors that contributed to their development [77]. Thus, the treatment of these conditions involves a combination of pharmacological treatments and personalized integrative medicine protocols [78,79]. These protocols are established according to the needs and preferences of each individual patient [80]. Previous studies have shown that they provide important benefits in several chronic conditions, especially oncological diseases [81,82]. Advanced medical technologies such as PAPIMI [83], Low-Level Laser Therapy (LLLT) [84], oxygen–ozone therapy [85], and neurofeedback [86] contribute significantly to improving the health status of patients with chronic conditions, as well as to reducing pain and inflammation.

From the perspective of the proposed conceptual model, the results demonstrate a high explanatory capacity and good statistical fit, confirmed by both the coefficients of determination and the model fit indices. In particular, patient satisfaction is explained to a proportion of 69% by the variables included in the model, highlighting the relevance of the analyzed factors in the context of advanced medical technologies integrated into integrative medicine. Among the tested hypotheses, perceived benefits and trust emerged as variables strongly associated with satisfaction, both showing significant positive associations. In contrast, experience and cost showed weaker associations, suggesting a secondary role. Hypotheses H1 and H5 were not supported. One possible explanation for the non-significant relationship between patient-reported outcomes and satisfaction is that patients may have evaluated their treatment experience not only through the clinical results obtained, but also through factors related to trust and the perceived benefits of the treatments. H5 was also not supported, even though previous studies reported a positive relationship between service quality and patient satisfaction. This may be specific to integrative medicine settings involving advanced technologies, where patients may place greater importance on treatment effectiveness and trust in the procedures than on traditional service quality dimensions. The results obtained illustrated that there is a strong relationship between the satisfaction experienced by patients and their intention to reuse the services provided. Previous studies conducted by Kim et al. [73] also highlighted a very strong relationship between these two variables, an aspect that was confirmed in the present research. As mentioned previously, it can be observed that the results obtained in this study differ from those reported by Woo and Choi [69]. The authors illustrated in their previous studies that service quality influences patient satisfaction. In the present study, it was observed that this variable did not have a strong influence on satisfaction. One possible explanation for these results may be related to the particularity of the services provided within integrative medicine protocols. In this context, perceived benefits, as well as trust in the services provided and in the medical staff, may have a much stronger impact compared to the quality of the services provided.

From the perspective of research limitations, it should be noted that the present study presents several methodological constraints that must be taken into account when interpreting the results. A first limitation concerns the relatively small sample size, which does not allow the extrapolation of the findings to the entire population under investigation. Furthermore, although there are only a limited number of clinics in Romania that provide treatments based on advanced medical technologies integrated into integrative medicine protocols, the research was conducted within a single private medical unit. This represents one of the main limitations of the study. However, it should be noted that, in Romania, this clinic is currently the only one that integrates all of these advanced technologies and is able to provide personalized treatments based on them. Most other clinics do not incorporate all of these advanced technologies within their treatment protocols, which would have made the analysis more difficult due to the lack of similarity between the procedures and services provided across clinics. Another limitation is related to the data collection process. For some patients, the questionnaire was completed within the clinic immediately after the treatment procedures. This aspect may have influenced patients’ responses, as some participants may have reported more positive perceptions and higher levels of satisfaction immediately after treatment. An additional limitation concerns the demographic profile of the respondents. Most participants were from urban areas, had higher levels of education, and had above-average incomes. This aspect may represent a source of selection bias. At the same time, it may limit the external validity and generalizability of the findings, especially in relation to patients from rural areas or those with lower income levels.

An additional aspect that should be considered when interpreting the findings concerns the profile of the respondents included in the study. Most participants came from urban areas and had relatively high levels of education and income. These characteristics may have influenced both patients’ expectations and their perceptions regarding technology-based integrative treatments. Therefore, it should be emphasized that the patients included in this research may have different perceptions and attitudes compared to the broader population. For this reason, the results cannot be fully generalized to the entire population under investigation. In general, individuals who choose integrative medicine protocols based on advanced technologies tend to be more open to innovative treatments. In many cases, these patients also have higher levels of trust in such procedures, which may explain their decision to use these therapies. Another important aspect is related to the socioeconomic status of the respondents. This factor may strongly influence both patients’ expectations and their perceptions regarding the outcomes that can be achieved through these treatment protocols. Therefore, the relatively high levels of satisfaction and acceptance identified in this study may not necessarily be reflected among individuals from rural areas or among patients with lower income levels. For this reason, caution should be exercised when generalizing these findings to the wider population.

Additionally, the study was carried out exclusively in Bucharest, which restricted the participation of patients from other counties and, consequently, reduced the geographical diversity of the sample. Another limitation refers to the fact that the proposed conceptual model included a relatively limited set of independent variables. The proposed model focused on a selected set of variables considered relevant based on the existing literature and the exploratory purpose of the study. However, additional factors not included in the present model may also influence patient satisfaction and intention to reuse such treatments. Moreover, the cross-sectional design of the study represents an important limitation. Because the data were collected at a single point in time, the proposed relationships cannot be interpreted as causal relationships. The SEM only reflects statistical associations between the analyzed variables.

Another limitation of the study, related to the way the questionnaire was designed, concerns the measurement of the two variables included in the proposed conceptual model. Patient satisfaction and intention to return were both measured as single-item constructs. These variables were evaluated using global assessment items because the study had an exploratory character. In addition, since a relatively large number of variables were included in the study, it was considered important to reduce the overall length of the questionnaire. The use of a single global item was intended to avoid confusion among respondents and to reduce respondent fatigue. Both patient satisfaction and intention to return were considered concepts that could be evaluated relatively easily by respondents at a general level. However, it should be taken into consideration that this type of measurement may provide lower psychometric robustness compared to multi-item scales. Furthermore, single-item measures may not fully capture the complexity of the variables analyzed in the study. Considering these aspects, future research should use multiple items for the evaluation of these variables. This approach may improve both measurement precision and construct reliability.

The finding that all respondents expressed an intention to recommend these treatments may reflect a high level of patient satisfaction. However, it should be interpreted with caution. Responses may have been influenced by social desirability bias and by the context in which the questionnaires were administered, namely within the clinical setting and shortly after treatment completion. These factors could have encouraged more favorable responses compared to those obtained in a fully neutral and externally administered environment. Future studies should consider the use of validated instruments for assessing social desirability bias, data collection outside the clinical setting, and enhanced anonymization procedures to further minimize potential response bias.

The relatively high R^2^ value (0.69) indicates strong explanatory power of the proposed model. However, because all variables were assessed through self-reported measures collected at a single time point, the potential influence of common method variance cannot be entirely excluded. Consequently, part of the observed explanatory power may reflect shared measurement context in addition to the actual relationships among constructs. Future studies employing longitudinal, multi-method, or multi-source designs are recommended to further validate these findings.

Although the study was approved by the ethics committee of the clinic where the research was conducted, a practice permitted under Romanian law (Legea 206/2004, Art. 9), the authors acknowledge that the absence of an external independent ethical review represents a limitation. Since the approving body and the data collection site belonged to the same institution, potential conflicts of interest cannot be entirely excluded. Future studies should seek evaluation by an independent ethics committee (e.g., affiliated with a university or national research ethics body) to further strengthen procedural rigor and transparency.

The research conducted in this study may provide added value and novelty both for medical professionals working in such clinics and for patients. From the perspective of contributions to medical staff, the study offers additional insights into the main factors associated with the level of satisfaction experienced by patients following treatments based on advanced medical technologies integrated into integrative medicine protocols. Thus, medical professionals working in clinics that provide such procedures can use the results obtained in order to organize their activities more efficiently and to improve the quality of services delivered to patients.

In addition, the SEM tested within this study can also be applied in other clinics operating in this field, offering a useful analytical framework for evaluating the factors associated with patient satisfaction. The study also provides valuable information that may support the improvement of management processes within integrative medicine clinics. Based on the results obtained, new personalized treatment schemes can be developed and adapted to different types of chronic diseases, while investments in medical equipment may be optimized according to actual needs and patient perceptions. From the patients’ perspective, this study may also provide significant value by highlighting the level of satisfaction among individuals with chronic diseases who have undergone at least one treatment based on advanced medical technologies integrated into integrative medicine protocols. Their opinions may represent an important reference point for other patients facing similar conditions who are considering the possibility of resorting to such treatments. Furthermore, the results of the study may contribute to increasing patients’ trust in these services by providing a solid basis for making informed medical decisions.

Regarding future research directions, it should be emphasized that the present study contributes both theoretically and practically. Nevertheless, there is a clear need for additional research to confirm or challenge the results of this study. In this regard, future research should include qualitative studies (for example, focus groups, in-depth interviews, etc.) conducted both among patients and among the medical staff who implement these treatments. Such studies would enable a deeper exploration of the topic and facilitate the identification of factors that prevent patients from accessing these treatments, the elements that could increase their attractiveness in the future, the ways in which these treatments could be improved, as well as the additional information that patients believe they need in order to increase their level of satisfaction. On the other hand, there is also a need for more extensive quantitative studies conducted on larger samples of respondents. It is important that such research be carried out both at the national and international levels in order to allow a stronger generalization of the conclusions and, potentially, to facilitate meaningful comparisons between regions or countries. Furthermore, future research would benefit from the implementation of longitudinal studies aimed at analyzing the evolution of patients’ perceptions over time. Such studies could highlight how patient satisfaction with these treatments is associated, in the long term, with their intention to use such treatments again if the need arises to treat the same condition or a new medical condition. Future research should also focus on conducting comparative analyses of the advanced medical technologies used within these treatments. Through such an approach, the perceived effectiveness, acceptability, and level of satisfaction associated with each technology could be examined separately. Moreover, future studies should integrate comparisons between the results obtained in quantitative research and the clinical evolution of patients. In this way, it would be possible to analyze in parallel patients’ satisfaction levels, the evolution of their medical tests, the stage of the chronic disease from which they suffer, and other relevant clinical variables. Another direction for future research would be the development of a clinical study aimed at comparatively analyzing the results obtained in two groups: patients with chronic diseases who undergo treatments based on advanced medical technologies integrated into integrative medicine protocols, and a control group. Such a study could provide a clearer understanding of both the clinical outcomes associated with these treatments and the level of satisfaction experienced by patients following these therapeutic interventions.

Considering the aspects presented above, it should be emphasized that this study provides added value both through its theoretical contributions and its practical applicability. It may represent a relevant reference point for the future development of treatments based on advanced medical technologies within the field of integrative medicine.

## 5. Conclusions

The integration of advanced medical technologies within integrative medicine protocols may contribute to the management of chronic conditions and may support a more patient-centered and holistic approach to care. These technologies may contribute to improving patients’ perceived treatment experience, while also being associated with higher levels of reported satisfaction among the respondents included in this study. Their use may facilitate access to innovative therapeutic interventions that may positively influence patients’ quality of life within the analyzed sample. At the same time, such approaches may support the management of complex conditions and help reduce their impact on patients’ daily functioning. The results also highlighted the importance of perceived benefits and trust in shaping patient satisfaction within integrative medicine settings. In addition, patient satisfaction was strongly associated with patients’ intention to reuse these treatments in the future. These findings should be interpreted in the context of the exploratory nature of the study, the cross-sectional design, and the fact that the data were collected from a single clinic.

## Figures and Tables

**Table 1:** Reliability statistics.

Cronbach’s Alpha	Cronbach’s Alpha Based on Standardized Items	N of Items
0.954	0.957	30

**Table 2:** Assessment of the measurement model.

Construct	Composite Reliability	Cronbach’s Alpha	AVE	Full Collinearity VIF
Patient-reported treatment outcomes	0.924	0.891	0.754	2.331
Perceived benefits	0.918	0.889	0.692	2.937
Patients’ prior experience	0.908	0.865	0.712	1.868
Patients’ trust in these treatments	0.938	0.917	0.751	2.068
Quality of services provided	0.934	0.914	0.707	1.935
Perceived cost	0.907	0.859	0.712	2.063
Patient satisfaction	1.000	1.000	1.000	2.207
Patient intention	1.000	1.000	1.000	2.588

**Table 3:** Hypothesis validation using the variance-based method.

No.	Hypotheses	β	*p*	Validation
H1	Patient-reported treatment outcomes → Patient satisfaction	0.03	=0.36	Not supported
H2	Perceived benefits → Patient satisfaction	0.5	<0.01	Supported
H3	Patients’ prior experience → Patient satisfaction	0.14	=0.04	Supported
H4	Patients’ trust in these treatments → Patient satisfaction	0.30	<0.01	Supported
H5	Quality of services provided → Patient satisfaction	0.05	=0.26	Not supported
H6	Perceived cost → Patient satisfaction	0.13	=0.05	Supported
H7	Patient satisfaction → Intention to return	0.65	<0.01	Supported

**Table 4:** Coefficients of determination (R^2^).

Patient Satisfaction	Intention to Return
0.69	0.42

**Table 5:** Model fit indices.

Indicators	Validation Criteria
Average path coefficient (APC) = 0.257	*p* < 0.001
Average R-squared (ARS) = 0.558	*p* < 0.001
Average adjusted R-squared (AARS) = 0.550	*p* < 0.001
Average block VIF (AVIF) = 1.995	Accepted if: the value generated is ≤5, Ideal ≤ 3.3
Average full collinearity VIF (AFVIF) = 2.250	Accepted if: the value generated is ≤5, Ideal ≤ 3.3
TenenhausGoF (GoF) = 0.664	Accepted if: the value generated is low ≥ 0.1, average ≥ 0.25, high ≥ 0.36
Sympson’s paradox ratio (SPR) = 1.000	Accepted if: the value generated is ≥0.7, Ideal = 1
R-squared contribution ratio (RSCR) = 1.000	Accepted if: the value generated is ≥0.9, Ideal = 1
Statistical suppression ratio (SSR) = 1.000	Accepted if: the value generated is ≥0.7
Nonlinear bivariate causality direction ratio (NLBCDR) = 1.000	Accepted if: the value generated is ≥0.7

## Data Availability

The original contributions presented in this study are included in the article. Further inquiries can be directed to the corresponding authors because the study data were collected within an institutional setting and are subject to ethical and data protection restrictions.
